# Self-constructed automated syringe for preparation of micron-sized particulate samples in x-ray microtomography

**DOI:** 10.1016/j.mex.2019.11.030

**Published:** 2020-01-10

**Authors:** Ralf Ditscherlein, Thomas Leißner, Urs A. Peuker

**Affiliations:** TU Bergakademie Freiberg, Agricolastraße 1, 09599 Freiberg, Germany

**Keywords:** Micron-sized particle sample preparation for XMT-analysis, Particle characterization, Sample preparation, X-ray microtomography, XMT, Automated syringe

## Abstract

In X-ray microtomography the sample has to meet special requirements regarding (1) mechanical stability (blurring), (2) geometry (FOV - field of view, rotational symmetry) and (3) composition (high attenuating phases). When analyzing micron-sized particulate material (e.g. powders), the particles in the FOV have to be (4) statistically representative and fixation (embedding matrix) becomes a critical issue due to segregation and agglomeration effects. The authors describe a self-constructed, low-cost automated syringe that allows controlling aspiration speed and suctioning volume. The carrier matrix is a wax structure that is shock frozen within a small polymeric tube. With this, the authors could successfully validate the method to determine particle size distributions (PSD). The described method is used in a related study by Ditscherlein et al. (2019).

• Low-cost automated syringe constructed with LEGO-parts and automatized with Arduino-microcontroller.
• Particle sample embedded within a shock-frozen wax matrix.
• Reproducibility successfully demonstrated by determining particle size distributions.

## Graphical abstract

**Figure:**
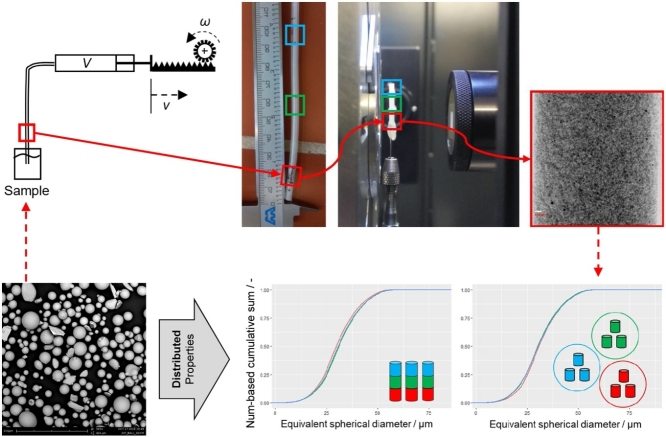

**Specifications Table**

**Table:** 

Subject Area:	Engineering
More specific subject area:	*Particle Characterization*
Method name:	*Micron-sized particle sample preparation for XMT-analysis*
Name and reference of original method:	*Please see bib-keys at the end of the document*
Resource availability:	**Hardware-part** https://store.wayneandlayne.com/products/bricktronics-shield-kit.html **LEGO Digital Designer** https://www.lego.com/de-de/ldd/download **LEGO Digital Designer File** FluidExtractionUnit.lxf **Arduino Script** FluidExtractionUnit.ino

## Method details

### Motivation

New application fields of particle technology, e.g. coatings, pharmaceuticals or electronic components require more and more highly defined particles in the lower micrometer range. In this case, the particle size distribution (PSD) is no longer sufficient to define the specifications alone. The particle system has to be described by the PSD, but also by further distributed properties, which are for instance the particle shape distribution or the particle composition distribution. Tomographic particle characterization is one key methodology to provide the data, required to quantify these specifications. The tomogram of a representative particle sample contains information on the size and shape of each individual particle. The data of X-ray absorption furthermore gives hints on the material properties and structure of intergrown or composite structures.

One central task in the tomography of a particle sample is the segmentation of the image data. When the particles in the sample are too close to each other, separating individuals can be challenging (one possible approach for segmentation algorithms for aggregated particles is given by Münch et al. [2]). On the other hand, oversegmentation can occur, when a particle is separated into two or more individuals during the image processing. In both cases, the physical sample preparation, which keeps the particles at a defined distance, is a key factor to minimize image segmentation errors.

**Fig. 1:**
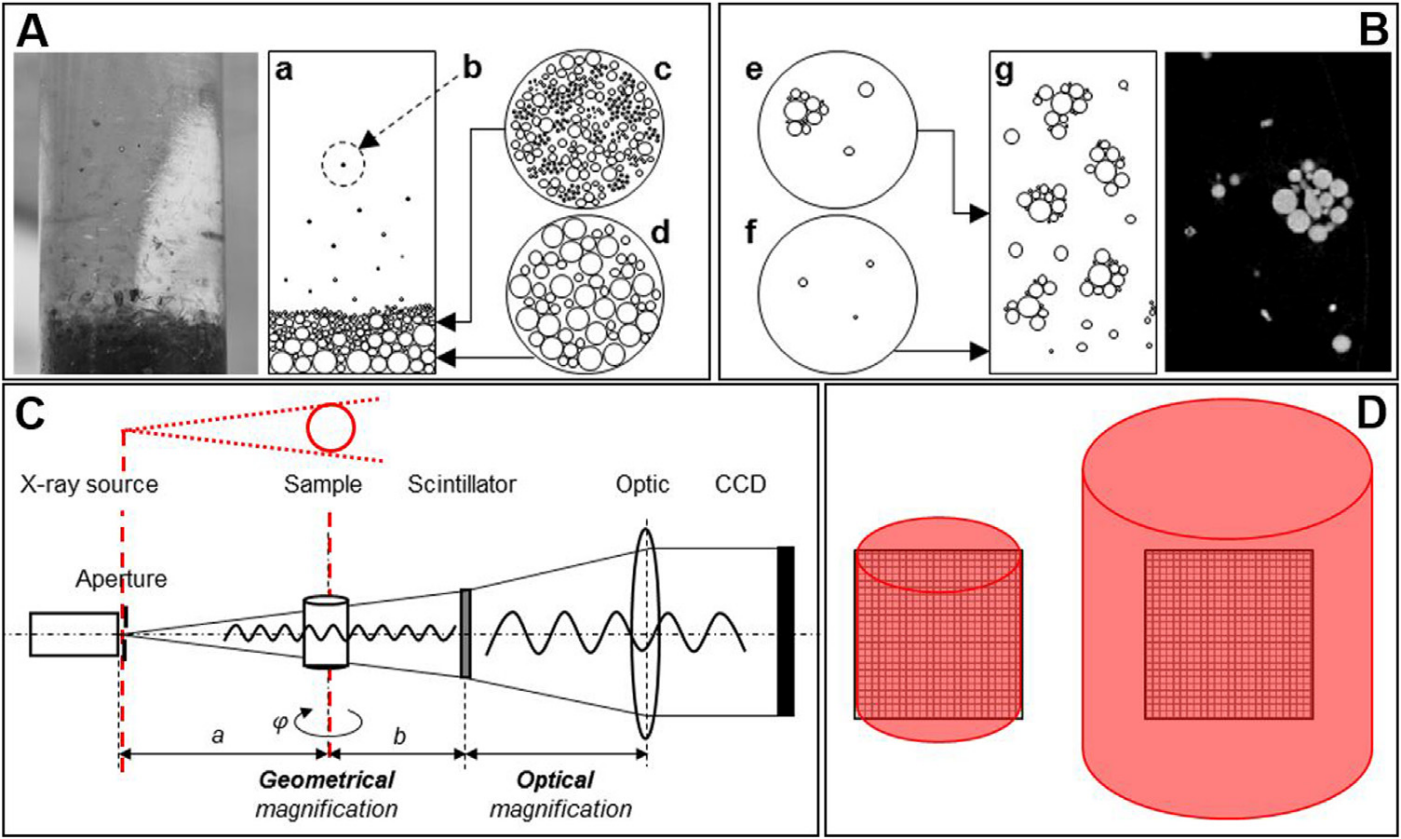
Segregation (A) and agglomeration (B) effects while embedding particulate samples in matrix, geometrical requirements for the sample: rotational symmetry (C) and appropriate sample size fitting to the field of view (D), adapted from [1].

The established techniques known from SEM, TEM or automated mineralogy (MLA) cannot be transferred to X-ray tomography, because they only have to provide a representative 2D-measuring plane. The 3D-sample preparation introduced here, is able to provide a homogeneous particle sample immobilized (minimization of segregation and agglomeration: Fig. 1-A, B) within a matrix material with low X-ray absorption that fits the required geometrical demands. This is (1) the rotational symmetry of the sample to ensure comparable X-ray-penetrated lengths (Fig. 1-C). (2) the sample has to fit to the desired field of view (FOV) to avoid region of interest tomography inside the sample that would increase exposure time (due to sample thickness and higher number of needed projections) and the probability of artefacts generated by material outside the FOV (Fig. 1-D).

To create such samples, there are already several syringes on the market starting at approx. 1300 €, but only for infusion. Dual systems for infusion and withdrawal starting at approx. 2300 €. They are offering multiple additional features and precision that is much too high for this application, which makes the equipment very expensive. The system described here reduces these costs by factor 10–15.

## Materials & methods

### Using wax as embedding matrix

Normal procedure for fixation of particles for volumetric scans is to embed them into a matrix that meets the requirements of the used measurement technique. In case of X-ray tomography, epoxy is a known standard adapted from 2D-methods like mineralogical analysis. It has a low X-ray attenuation coefficient and is stable at room temperature. In case of particles in the lower micrometer range, the settling velocity of the particles is not negligible. Due to the hardening time of the resin, segregation leads to a different composition of vertical sections. Direct particle contact can lead to a challenging segmentation procedure, especially in case of irregular shapes.

In recent studies, gel-like matrixes have been used for particle sample preparation that are stable at room temperature (e.g. Agar investigated by [3], but not good machinable. The focus of this study is to create a sample that is stable over a long period of time and is good machinable (e.g. for correlative analysis where sub-samples have to be cut out to reduce the FOV or to prepare 2D sections). Histological wax is an alternative that is known from biological sample preparation due to the good

compatibility of matrix and investigated structures. Also positive is a negligible shrinkage rate while hardening, good cutting properties and a low X-ray attenuation coefficient that is comparable to resin. A good summary of miniaturized alternatives to conventional sample preparation techniques for solid samples is given by Pena-Pereira [4]. A good summary of existing methods for biological sample preparation and their applications in X-ray microtomography is given by Strotton [5]. In 3D particle analysis, wax embedding methods are rarely mentioned. One example is given by Van Meel et al. [6], where wax is used as binder for tablets.

**Fig. 2:**
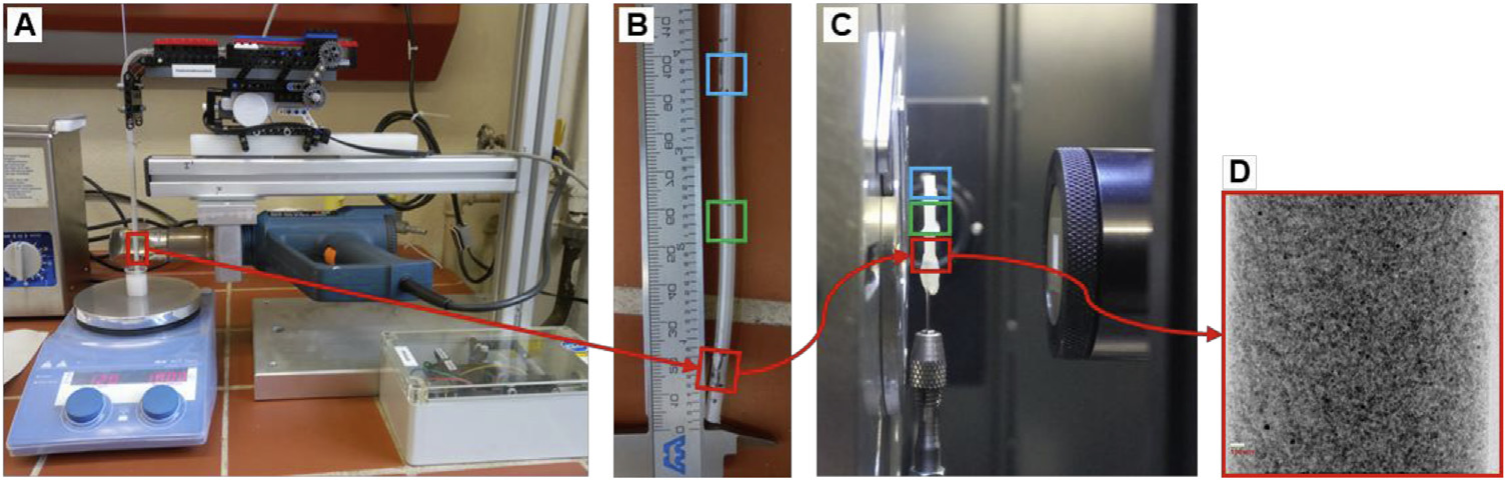
Sample preparation rack with extraction unit and heating gun (A), polymeric tube with solidified wax after shock freezing (B), sample stack with sub-samples from 3 different positions (top, middle, bottom) (C) and projection image from one sub-sample from sample stack (D).

Only using wax does not solve the problem of segregation. For this, the wax is additionally shock frozen within a small polymeric tube (Fig. 1-E, F). The experimental setup with a mounted validation sample is shown in Fig. 2.

### Specifications

The authors used a histological wax from PothHille (PHC9061) with a melting point between 59 and 61 °C. The silicone tube has an inner diameter of 2 mm, which corresponds to the requested sample size for the tomographic measurements (X-ray microscopy system ZEISS Xradia VERSA 510, 2048 pixel detector, camera binning 2, 80 keV, 7 W).

### Automated syringe

**Fig. 3:**
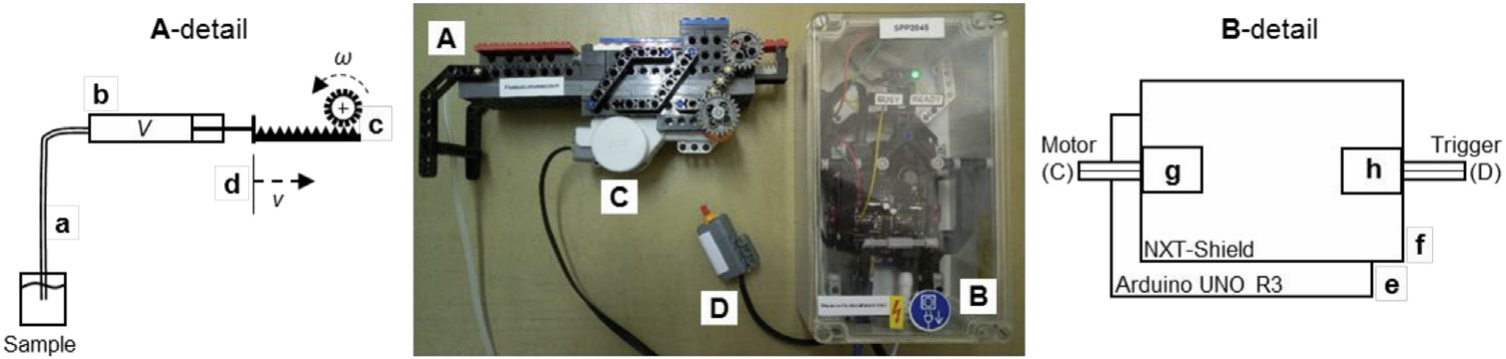
Fluid extraction unit (A) with controller (B), motor (C) and trigger (D), A-detail: flexible tube (a) mounted on a syringe (b) with a dynamic suction volume V which is increased by transforming rotational motion of a gear (c) in translation (d) with the speed v, B-detail: Arduino UNO R3 as basis (e) with a NXT-Shield (f) as extension to connect LEGO-motor (g, C) and trigger (h, D).

**Fig. 4:**
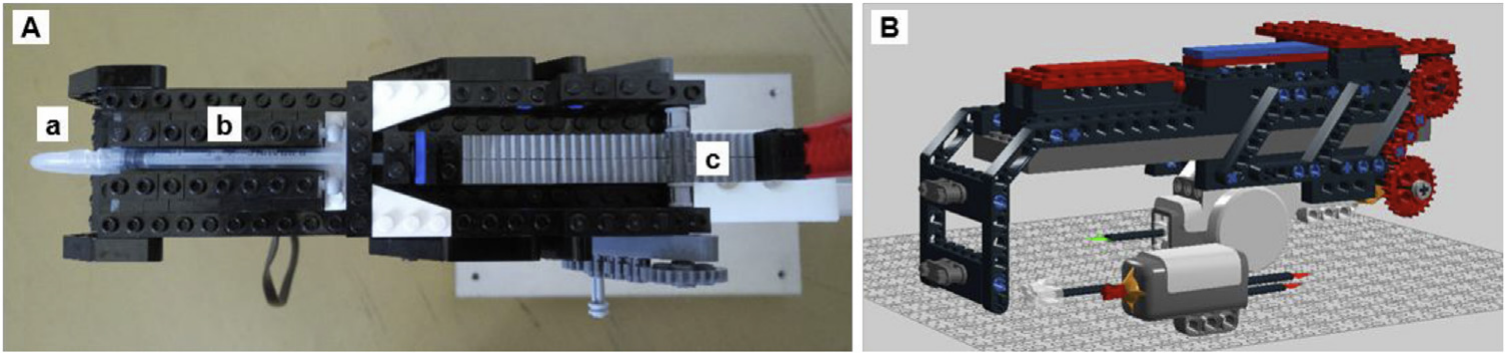
Syringe mounting (A) with tube connection (a), syringe (b) and rack (c); 3D construction model in LEGO Digital Designer (B).

The automated syringe is shown in Fig. 3. For the construction, LEGO Digital Designer^1^ was used. To connect the Arduino-Board (AB) to the LEGO-NXT motors and sensors, a circuit board from Wayne & Lane^2^ was used. The high torque of the NXT-motors can move the piston of the syringe accurately and with low voltages of the AB, which saves additional circuit boards and power sources. The AB offers the chance to program individual movement paths, adapted to the specific needs of the sampled material, e.g. a controlled start-up to prevent abrupt changes of motion, which can cause unwanted mechanical stress. The LEGO-parts are listed in Table 1. Fig. 4-A shows the assembled syringe mounting with tube connection (a), syringe (b) and rack (c). Fig. 4-B shows the 3D construction model from LEGO Digital Designer. The corresponding file and the Arduino-Script can be found in the supplemental material.^3^

**Table 1:** LEGO part list with brick number, name and quantity.

Brick	Name	Quantity
300526	BRICK 1 × 1	2
300426	BRICK 1 × 2	1
300326	BRICK 2 × 2	4
654126	TECHNIC BRICK 1 × 1	6
370026	TECHNIC BRICK 1 × 2, Ø4.9	1
3200026	BRICK 1 × 2 M. 2 HOLES Ø 4,87	1
370126	TECHNIC BRICK 1 × 4, Ø4,9	6
389426	TECHNIC BRICK 1 × 6, Ø4,9	2
370226	TECHNIC BRICK 1 × 8	2
273026	TECHNIC BRICK 1 × 10 Ø4.9	2
389526	TECHNIC BRICK 1 × 12, Ø4,9	5
302421	PLATE 1 × 1	2
302326	PLATE 1 × 2	3
379426	PLATE 1 × 2 W. 1 KNOB	16
243123	FLAT TILE 1 × 4	6
306821	FLAT TILE 2 × 2	3
306823	FLAT TILE 2 × 2	7
302226	PLATE 2 × 2	1
371026	PLATE 1 × 4	2
366626	PLATE 1 × 6	5
302026	PLATE 2 × 4	2
4514845	PLATE 1 × 12	6
303126	PLATE 4 × 4	5
303521	PLATE 4 × 8	2
428226	PLATE 2 × 16	1
428221	PLATE 2 × 16	1
4211114	PLATE 6 × 10	3
4210720	PLATE 6 × 14	3
4180508	RIGHT PLATE 2 × 3 W/ANGLE	1
4180536	LEFT PLATE 2 × 3 W/ANGLE	1
4297185	CABLE 0,5 M	2
4296929	Push sensor	1
4297008	Tacho Motor	1
4142822	TECHNIC 3 M BEAM	1
3227126	TECHNIC ANGULAR BEAM 3 × 7	2
662926	TECHNIC ANGULAR BEAM 4 × 6	4
4107578	DOUBLE ANGULAR BEAM 3 × 7 45°	2
663226	TECHNIC LEVER 3 M	2
4211573	1/2 BUSH	2
4211483	CONNECTOR PEG W. KNOB	2
4142865	2M CROSS AXLE W. GROOVE	1
273621	BALL W. CROSS AXLE	2
4129886	CONNECTOR PEG	23
4225927	CONNECTOR PEG/CROSS AXLE	13
4211622	BUSH FOR CROSS AXLE	2
4514553	CONNECTOR PEG W. FRICTION 3 M	3
4211086	CROSSAXLE 3 M WITH KNOB	4
4210810	DOUBLE CROSS BLOCK	2
370726	CROSS AXLE 8M	1
373726	CROSS AXLE 10M	1
4120102	GEAR WHEEL T = 8, M = 1	5
374323	TOOTHED BAR M = 1, Z = 10	6
4142825	GEAR WHEEL Z24	2
393826	PLATE 1 × 2 (ROCKING)	3
393726	ROCKER BEARING 1 × 2	3

## Method validation

**Fig. 5:**
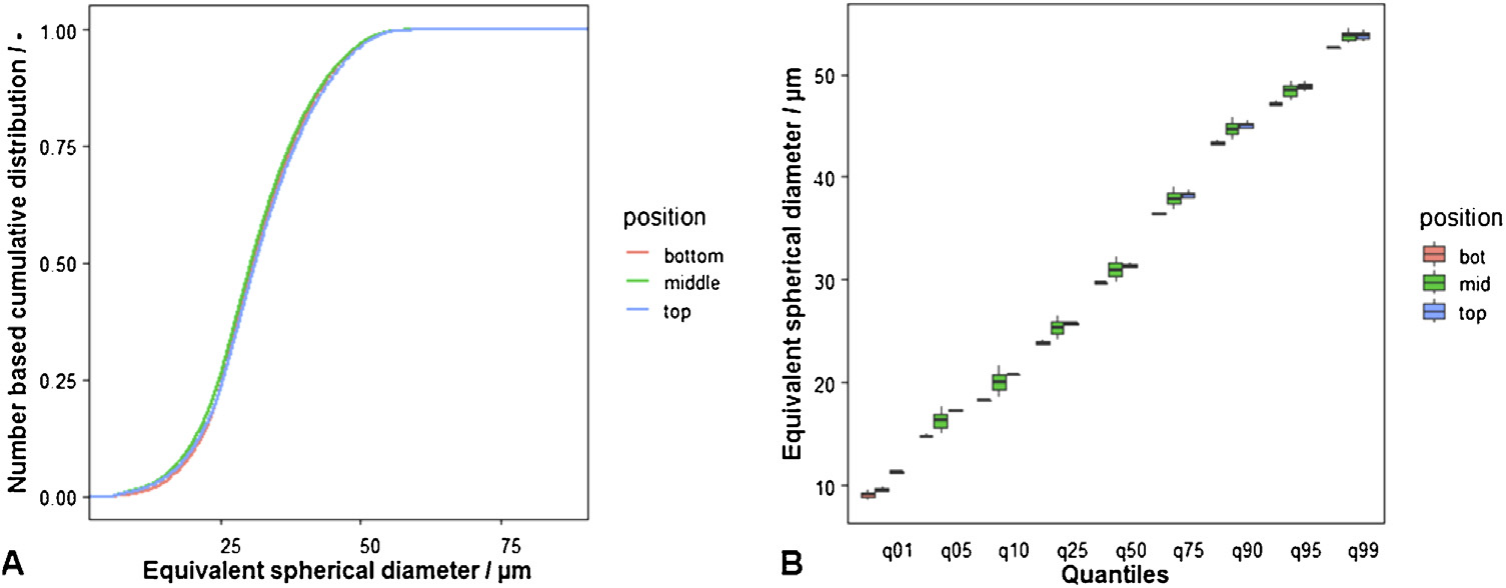
Number based cumulative distribution of the equivalent spherical diameter (ESD) of a spherical particle sample (5…50 μm) for 3 different extraction points from the sample tube (A), quantiles of the ESD at the same positions for variation-analyses (B).

As idealized sample, a spherical soda-lime glass-particle system (SiLibeads, type S from Sigmund Lindner) with particle sizes below 50 μm was chosen (72.3% SiO2, 13.3% Na2O, 8.9% CaO, 4.0% MgO, 1.5% others). The number based cumulative distribution at sample positions top, middle and bottom (with regard to their extraction height from the polymeric tube) and the characteristic quantiles show no significant differences (data basis: 3 replicates for each extraction position). Detailed results are summarized in Table 2. Number based cumulative distributions and summarizing box-plots of all extraction heights are shown in Fig. 5. More details can be found in Ref. [1].

**Table 2:** Aggregated results particle size distribution (average, standard deviation) from all measurements; For experiments tube extraction height-validation (TEH) and extraction point-validation (EP) with given target particle concentration and average number of particles N per experiment.

Quantile	avg / μm			stddev / μm		
Exp.	TEH		EP	TEH		EP
Conc.	10%	15%	10%	10%	15%	10%
0.01	10.3	10.0	10.0	0.5	2.4	1.0
0.05	15.8	15.8	16.1	0.8	2.5	1.3
0.10	19.2	19.7	19.7	1.0	1.6	1.4
0.25	24.8	25.0	25.0	0.9	1.0	1.1
0.50	30.6	30.6	30.7	0.8	1.0	1.0
0.75	37.6	37.6	37.5	0.7	1.2	1.0
0.90	44.6	44.4	44.4	0.6	1.1	1.0
0.95	48.3	48.1	48.1	0.6	0.9	1.0
0.99	53.5	53.4	53.4	0.6	0.7	0.8
N (avg)	25580	35220	24770

## Declaration of Competing Interest

The authors certify that they have NO affiliations with or involvement in any organization or entity with any financial interest (such as honoraria; educational grants; participation in speakers’ bureaus; membership, employment, consultancies, stock ownership, or other equity interest; and expert testimony or patent-licensing arrangements), or non-financial interest (such as personal or professional relationships, affiliations, knowledge or beliefs) in the subject matter or materials discussed in this manuscript.

## Footnotes

1 https://www.lego.com/de-de/ldd/download.

2 https://store.wayneandlayne.com/products/bricktronics-shield-kit.html.

3 FluidExtractionUnit.lxf, FluidExtractionUnit.ino.

## References

[1] Ditscherlein R., Leißner T., Peuker U.A. (2019). Preparation techniques for micron-sized particulate samples in X-ray microtomography. Powder Technol..

[2] Münch B., Gasser P., Holzer L., Flatt R. (2006). FIB-nanotomography of particulate systems - Part II: particle recognition and effect of boundary truncation. J. Am. Ceram. Soc..

[3] Ashrafi K., Tang Y., Britton H., Domenge O., Blino D., Bushby A.J. (2017). Characterization of a novel intrinsically radiopaque Drug-eluting Bead for image-guided therapy: DC Bead LUMI^TM^. J. Control. Release.

[4] Pena-Pereira F. (2014). Miniaturized alternatives to conventional sample preparation techniques for solid samples. De Gruyter Open.

[5] Strotton M.C., Bodey A.J., Wanelik K., Darrow M.C., Medina E., Hobbs C. (2018). Optimising complementary soft tissue synchrotron X-ray microtomography for reversibly-stained central nervous system samples. Sci. Rep..

[6] Van Meel K., Smekens A., Behets M., Kazandjian P., Van Grieken R. (2007). Determination of platinum, palladium, and rhodium in automotive catalysts using high-energy secondary target X-ray fluorescence spectrometry. Anal. Chem..

